# The Effect of Different Glycaemic States on Renal Transplant Outcomes

**DOI:** 10.1155/2016/8735782

**Published:** 2016-12-07

**Authors:** Angela Sheu, Barbara Depczynski, Anthony J. O'Sullivan, Grant Luxton, George Mangos

**Affiliations:** ^1^Department of Endocrinology, Prince of Wales Hospital, Randwick, Sydney, NSW, Australia; ^2^Prince of Wales Clinical School, UNSW Medicine, Randwick, Sydney, NSW, Australia; ^3^Department of Endocrinology, St George Hospital, Kogarah, Sydney, NSW, Australia; ^4^St George & Sutherland Clinical School, UNSW Medicine, Kogarah, Sydney, NSW, Australia; ^5^Department of Nephrology, Prince of Wales Hospital, Randwick, Sydney, NSW, Australia; ^6^Department of Nephrology, St George Hospital, Kogarah, Sydney, NSW, Australia

## Abstract

*Background*. Optimal glycaemic targets following transplantation are unknown. Understanding the impact of DM and posttransplant diabetes mellitus (PTDM) may improve patient and graft survival in transplant recipients.* Aim*. To determine the perioperative and one-year outcomes after renal transplantation and whether these outcomes are affected by preexisting DM, PTDM, or glycaemia during transplant admission.* Method*. Adult recipients of renal transplants from a single centre over 5.5 years were retrospectively reviewed. Measured outcomes during transplant admission included glycaemia and complications (infective complications, acute rejection, and return to dialysis) and, at 12 months, glycaemic control and complications (cardiovascular complication, graft failure).* Results*. Of 148 patients analysed, 29 (19.6%) had DM and 27 (18.2%) developed PTDM. Following transplantation, glucose levels were higher in patients with DM and PTDM. DM patients had a longer hospital stay, had more infections, and were more likely return to dialysis. PTDM patients had increased rates of acute rejection and return to dialysis. At 1 year after transplant, there were more cardiovascular complications in DM patients compared to those without DM.* Conclusions*. Compared to patients without DM, patients with DM or PTDM are more likely to suffer from complications perioperatively and at 12 months. Perioperative glycaemia is associated with graft function and may be a modifiable risk.

## 1. Introduction

Hyperglycaemia is frequently seen following renal transplantation [1], due to the effects of immunosuppressant regimens, particularly glucocorticoids, on glucose metabolism, and the presence of preexisting diabetes. There is limited evidence for a specific postoperative blood glucose (BG) range following surgery other than coronary artery bypass grafting. In the absence of specific data, standard perioperative targets of 5–10 mmol/L during the perioperative period are usually recommended [2]. However, these perioperative glucose targets may not be appropriate in patients with end stage renal failure, which is a catabolic and insulin resistant state [3]. Specifically, appropriate glycaemic targets during the transplant admission are not clear. During the transplant admission, there is evidence of harm from both hypoglycaemia [4] and hyperglycaemia [5]; how these relationships are modified by pretransplant diabetes status is not clear. Understanding any impact of glycaemia on outcomes may provide support to determine appropriate glucose targets.

The aim of this study was to determine perioperative and one-year outcomes after renal transplantation and whether these outcomes are affected by preexisting DM (DM) or posttransplant diabetes mellitus (PTDM) or, alternatively, by glycaemia during the transplant admission.

## 2. Materials and Methods

### 2.1. Subjects

We performed a retrospective review of all patients who underwent kidney transplantation at a single tertiary referral centre for all local renal transplants from January 2009 until June 2014. Eligible patients were adults with at least one year of follow-up data available. Results were adjudicated at one year. The study was approved by the local hospital ethics committee.

All patients undergoing renal transplantation had point of care capillary blood glucose monitoring performed preprandially and before bed for the first 72 hours after renal transplant. Given the pattern of hyperglycaemia usually seen with glucocorticoids with relatively unaffected fasting BG with peak BG seen in afternoon or evening [6], fasting BG and peak BG for days 1–3 after transplant were collected.

### 2.2. Definitions

DM: listed as a pretransplant comorbidity
PTDM: any of the following present: management instituted based on hyperglycaemia and required beyond the first 3 months after transplant, hyperglycaemia documented according to current ADA criteria, or if defined by the treating physician on follow-up
Acute rejection (AR): biopsy-proven rejection (Banff criteria)
Cardiovascular (CV) complication: composite of acute myocardial infarction, cerebrovascular accident, or peripheral vascular disease requiring intervention
Infection: positive culture or treatment with antibiotics instituted

### 2.3. Statistics

Statistics was performed by SPSS v22. ANOVA was performed for continuous variables. For other variables, the chi-square test or *t*-test was used as appropriate. Given that this was a retrospective study and all results are exploratory, corrections for multiple comparisons were not made. Correlation testing was determined by Pearson's or Spearman's coefficients as appropriate. Where binary logistic regression was performed, stepwise analysis was performed, known risk factors for delayed graft function as covariates.

## 3. Results

In total, 148 patients were analysed: 29 patients (19.6%) had preexisting DM and 27 patients (18.2%) developed PTDM. Of those with preexisting DM, 93.1% had type 2 DM and the remaining 6.9% had type 1 DM. Standard immunosuppression was initial basiliximab and methylprednisolone as well as ongoing prednisolone, mycophenolate, and either tacrolimus (79%) or cyclosporin (22%) based on the treating clinician's decision, in line with the recommendations at the time of transplantation. Patients assessed by the treating clinician to be at higher risk of rejection were treated with additional rabbit antithymocyte globulin (62%) at induction. There were no differences in type of immunosuppression regimens between the 3 groups (no DM, DM, and PTDM). The cumulative glucocorticoid dose in the first week was not different across the 3 groups (853 mg in no DM, 888 mg in DM, and 914 mg in PTDM); however, on day 7 after transplant, the mean prednisone dose was higher in those who developed PTDM (74 mg versus no DM, 30 mg, *p* < 0.05). Dose of prednisone at latter time points was not collected.

### 3.1. Baseline Characteristics

Baseline characteristics of patients are shown in Table 1. DM patients were older, more obese, and more likely to have had a prior vascular event. They were also more likely to be treated with a statin or any antihypertensive medication. Mean HbA1c was 54 ± 10 mmol/mol (7.1 ± 1.4%) prior to transplant, which was higher than those without DM (36 ± 3 mmol/mol [5.4 ± 0.5%], *p* < 0.001). Of those who developed PTDM, 14 (52%) were diagnosed during the initial transplant admission and the remainder 1 to 6 months after the transplant. PTDM patients had higher triglyceride levels at baseline compared to those without DM (2.6 mmol/L versus 1.7 mmol/L, *p* < 0.01).

Fasting glucose levels taken on admission for transplant were higher in DM patients compared to those without DM (6.8 ± 1.8 mmol/L versus 5.1 ± 0.9 mmol/L, *p* < 0.05) (Table 2). Fasting and peak BG on days 1, 2, and 3 following transplant were significantly different across the 3 groups. Hypoglycaemia (BG < 3.5 mmol/L) only occurred on 5 occasions days 1–3 postoperatively and so was not analysed further. There were no episodes of severe hypoglycaemia.

### 3.2. Outcomes of Transplant Admission

Following transplantation, DM patients had a longer hospital stay (16.9 ± 9.7 versus 13 ± 5.6 days, *p* < 0.01), had more infective complications during that admission (34.5% versus 13%, *p* < 0.01), and were more likely to return to dialysis (48.3% versus 19.6%, *p* < 0.01) as compared to those with no DM (Table 3). There was no difference in the rate of acute rejection (AR) in patients with DM as compared to those without DM (13.8% versus 7.6%, *p* = NS). PTDM patients had increased rates of returning to dialysis (51.9% versus 19.6%, *p* < 0.01) and AR (25.9% versus 7.6%, *p* < 0.05) but no differences in their length of stay or infective complications compared to those without DM (Table 3).

Any relationship between risk of infection and prior glycaemic control could not be assessed as <50% of the whole cohort had an HbA1c result available from the year prior to transplant. In DM patients, there was a nonsignificant increase in the HbA1c in those who had an infective complication compared to those without (60 ± 9 mmol/mol [7.6 ± 1.2%] versus 53 ± 12 mmol/mol [7.0 ± 1.4%], *p* = 0.25). Although HbA1c is less reliable in this cohort and this is underpowered, there is a suggestion of a trend towards worse glycaemic control in those with infective complications. There was no correlation with fasting BG taken at admission for transplantation nor with fasting BG or peak BG on day 1, 2, or 3 after transplant and occurrence of infection. There was no relationship between return to dialysis and fasting glucose just prior to transplantation. For the whole cohort, return to dialysis during transplant admission correlated with fasting and peak BG on day 2 and 3 postoperatively. Binary logistic regression was performed using a stepwise analysis. When adjusted for type of donor (deceased versus live) and HLA mismatching (both known factors for delayed graft function and return to dialysis), of the fasting and peak BG on days 1–3, only fasting BG on day 3 remained significant (OR 1.63, 95% CI 1.11–2.39, *p* = 0.013). There was no relationship between days 1–3 fasting or peak BG and AR during transplant admission. In those who developed PTDM as compared to those without diabetes, days 1, 2, and 3 fasting and peak BG were significantly higher (Table 2).

### 3.3. Outcomes at 12 Months following Transplant

At follow-up, glycaemia remained significantly higher in those with diabetes. At 12 months, mean HbA1c for DM patients was 61 ± 14 mmol/mol (7.7 ± 1.8%; DM versus no DM, *p* < 0.01), 48 ± 5 mmol/mol (6.5 ± 0.7%) for patients with PTDM (versus no DM, *p* < 0.05), and 38 ± 4 mmol/mol (5.6 ± 0.6%) for those patients without DM (Table 4). There were no differences in serum lipids except for lower total cholesterol in the DM patients compared to those without DM (4.5 versus 5.1 mmol/L, *p* < 0.05) which could be accounted for by lipid treatment.

At 12 months after transplant, patients with DM had more cardiovascular complications (20.7% versus 4.3%, *p* < 0.05) compared to those without diabetes (Table 4). In those with DM, cardiovascular events were associated with age and smoking status but not with HbA1c, prior history of a vascular event, BMI, or pretransplant lipids. There were no differences in eGFR, infection, or mortality. Graft loss censored for death (return to dialysis or death) at one year was not significantly different between the 3 groups and did not correlate with days 1–3 BG. At 12 months, there were no differences in any of the measured outcomes in those with PTDM compared to those without DM.

## 4. Discussion

This retrospective study has shown that patients with DM and PTDM have worse perioperative outcomes after renal transplantation compared to those without DM. These patients have increased rates of return to dialysis after transplant, and this need was related to BG control during transplant admission. DM was associated with increased risk of infection during transplant admission, although this risk was unrelated to glycaemia. PTDM patients more frequently experienced rejection during the transplant admission. The occurrence of AR was unrelated to BG levels. BG levels were highest in those with DM; those who developed PTDM also had BG levels significantly higher than those without DM. At 12 months, the only difference in outcomes was an increased number of cardiovascular events in those with DM.

Graft survival is reduced where delayed graft function (DGF) has occurred [7]. Our results confirm that DM is a risk factor for DGF [8, 9] and suggest that pretransplant hyperglycaemia contributes to pathogenesis of DGF, possibly due to increased ischaemic-reperfusion injury [8]. In our analysis, we controlled for known risk factors for DGF (such as type of donor and HLA mismatching) and found that fasting BG on day 3 remained significant. This suggests that hyperglycaemia immediately after transplant may also represent a potentially modifiable risk factor for DGF. We were unable to collect data on other known risk factors, such as donor age and cold ischemia time. A single trial of tight BG control after transplant (with intensive intravenous insulin to target BG 3.9–5.5 mmol/L) compared to subcutaneous insulin (target BG 3.9–9.9 mmol/L) did not show a benefit but may have been confounded by high rate of hypoglycaemia which was associated with an increase in acute rejection [4, 10].

Inflammation may also participate in the development of DGF [7]. Both DM and PTDM are inflammatory states [11] and hence increase the risk of DGF. However, hyperglycaemia may reflect rather than cause the inflammation. Further, the degree of hyperglycaemia may not reflect the intensity of the inflammation. Alternatively, as only day 3 FBG remained significant after adjustment for covariates, hyperglycaemia may reflect reduced glycosuria as a consequence of DGF. The glycaemic impact of renal handing of glucose by the transplanted kidney is unknown.

Given the relationship between hyperglycaemia and infection risk in other inpatient groups [12] we hypothesised a relationship between peak BG and risk of infection, as mean BG is not likely to adequately reflect the degree of hyperglycaemia secondary to glucocorticoids. We found that postoperative daily fasting or peak BG was unrelated to infection arising during transplant admission, which is in keeping with prior studies which did not show a relationship between mean BG during transplant admission and infection by 30 days [13] or by 12 months [14]. The increased risk of infection seen only in those with DM may relate to other factors such as prior glycaemic control, the chronic inflammatory milieu of DM, or obesity.

Our results are consistent with other groups that hyperglycaemia is common during the transplant admission [15] and that inpatient hyperglycaemia is a predictor for development of PTDM [5]. Pretransplant fasting glucose and HbA1c were elevated in those who developed PTDM, suggesting that PTDM is driven by both transplant-specific and transplant-independent factors and that adequate pretransplant screening, combined with posttransplant hyperglycaemia, may predict those who develop PTDM. Pretransplant HbA1c was limited to <50% of patients, limiting its usefulness in identifying any meaningful relationship between longer term glycaemic variability and outcomes. Future studies to investigate the diagnostic utility of combining pretransplant glycaemic control and posttransplant glycaemia are warranted.

Although tight glycaemic control after transplant may not be useful for preventing infection, a single trial has shown that modest glycaemic control using isophane insulin was able to reduce the risk of PTDM in kidney recipients [16]. The increased risk of AR seen in patients who developed PTDM is also consistent with prior findings [17]. We found no relationship between perioperative glycaemia and AR, supporting the supremacy of preventing rejection rather than modifying diabetogenic immunosuppressant regimens as the primary strategy to prevent PTDM [18]. Although PTDM has been associated with poorer outcomes in early studies [1], our results at 12 months are consistent with more recent findings that outcomes of those with PTDM are similar to those without diabetes [19, 20], although this is limited by short follow-up and small numbers.

Cardiovascular events were more frequent following transplant in those with DM compared to those without DM and PTDM. This was despite greater use of antihypertensive therapy and statin therapy at time of transplantation and equivalent lipid targets, suggesting a limitation to cardiovascular risk prediction by traditional factors [21]. Although this is a retrospective study and is not powered for cardiovascular outcomes, this result does highlight the need for future studies to better address the question of the ideal glycaemic target, both for inpatients and in the longer term, given the coexistence of both established microvascular disease and high risk for macrovascular disease. PTDM patients did not have increased cardiovascular disease at one year, suggesting that that cardiovascular risk factor management should be in keeping with those with CKD [22].

Our study is limited by a number of factors. The data is observational and retrospective, limiting conclusions on causality. We included a number of criteria for PTDM as formal screening for PTDM with an oral glucose tolerance test was not routine in our institution. We therefore included patients in whom treatment for hyperglycaemia was instituted or a physician diagnosis of PTDM recorded, on the basis that elevated glucose levels had been measured elsewhere, in order to maximise the number of diagnosed patients, although there could be selection bias. Glycaemic management and other cardiovascular risk factor management were individualised, which may have also affected outcomes that could not be quantified. BG measurements were performed by point of care reading, which can be affected by a number of posttransplant factors. However, our study strengths include frequent BG testing in all patients during the transplant admission, specifically with separate analysis of fasting and peak BG and not just mean BG, allowing for more detailed examination of the effect of inpatient hyperglycaemia.

## 5. Conclusions

Our study is a detailed examination of outcomes after renal transplantation and has shown that these outcomes vary according to the presence of preexisting or posttransplant diabetes and transplant admission glycaemia. The presence of diabetes is a risk factor for infection during transplant admission. Perioperative glycaemic control is associated with return to dialysis and may represent a modifiable risk. Further studies to identify the effect of intervention and appropriate glucose targets in this cohort are warranted.

## Figures and Tables

**Table 1:** Patient characteristics prior to transplantation.

Characteristics	No DM	DM	PTDM
Patients number (%)	92 (62)	29 (19.6)	27 (18.2)
Age^#^ (yrs)	47.9 ± 13.6	57.6 ± 5.8^*∗*^	55.1 ± 8.9^*∗*^
Male (%)	63	57	52
Caucasian (%)	81.5	65.5	77.8
BMI^#^(kg/m^2^)	25.3 ± 5.2	31.8 ± 4.8^*∗*^	24.2 ± 9.1^∧^
RRT > 12 months prior to transplant (%)	60.9	62.1	63
Current smoking (%)	3.3	6.9	11.1
Prior vascular event (%)	17.4	44.8^*∗*^	22.2
Prior transplant (%)	13	0	18.5
Deceased donor (%)	65.2	82.8	88.9
HLA mismatch ≥ 4/6 (%)	38.0	37.9	48.1
HbA1c (%)^#^	5.4 ± 0.5^##^	7.1 ± 1.4^*∗*^	6.0 ± 0.3^##^
HbA1c (mmol/mol)	36 ± 3	54 ± 11^*∗*^	42 ± 2.1
Total cholesterol (mmol/L)^#^	4.3 ± 1.1	4.1 ± 1.4	4.3 ± 1.1
Triglycerides (mmol/L)^#^	1.7 ± 0.8	2.1 ± 1.5	2.6 ± 2.1^*∗*^
HDL (mmol/L)^#^	1.2 ± 0.4	1.1 ± 0.5	1.1 ± 0.5
LDL (mmol/L)^#^	2.3 ± 0.9	2.0 ± 0.9	2.2 ± 0.9
Use of statin (%)	34.3	68.2^*∗*^	60.0^*∗*^
Use of ACEI (%)	17.6	18.2	25.0
Use of ARB (%)	16.2	27.3	31.6
Use of any antihypertensive (%)	40.3	76.2^*∗*^	52.6

^#^Expressed as mean ± SD; ^##^results missing for 50% of group; ^*∗*^ *p* < 0.05 compared to No DM; ^∧^ *p* < 0.05 compared to DM.

No DM: patients without DM, DM: patients with DM prior to transplantation, PTDM: posttransplant diabetes mellitus, BMI: body mass index, RRT: renal replacement therapy, HbA1c: glycosylated haemoglobin, ACEI: angiotensin converting enzyme inhibitor, and ARB: angiotensin receptor blocker.

Statistical analysis using ANOVA, chi-squared, and *t*-tests.

At baseline, DM patients were older, more obese, and more likely to have had a prior vascular event. PTDM patients had higher triglyceride levels compared to those without DM.

**Table 2:** Point of care capillary blood glucose levels.

	No DM	DM	PTDM
Day 0 prior to transplant			
Fasting	5.1 ± 0.9	6.8 ± 1.8^*∗*^	5.9 ± 1.0
Day 1 after transplant			
Fasting	7.1 ± 1.9	13.6 ± 5.5^*∗*^	9.3 ± 2.8^*∗*^
Peak	8.1 ± 1.8	14.7 ± 4.4^*∗*^	10.9 ± 2.3^*∗*^
Day 2 after transplant			
Fasting	6.1 ± 1.4	10.4 ± 2.3^*∗*^	7.9 ± 2.6^*∗*^
Peak	8.1 ± 1.6	15.3 ± 5.1^*∗*^	10.1 ± 3.0^*∗*^
Day 3 after transplant			
Fasting	5.4 ± 1.0	10.0 ± 3.4^*∗*^	6.7 ± 2.2^*∗*^
Peak	7.8 ± 2.0	16.0 ± 3.9^*∗*^	11.1 ± 3.4^*∗*^

Results are mean ± SD (mmol/L). ^*∗*^ *p* < 0.05 compared to No DM.

No DM: patients without DM, DM: patients with DM prior to transplantation, and PTDM: posttransplant diabetes mellitus.

Statistical analysis using ANOVA.

Prior to transplantation, blood glucose levels were higher in those with preexisting DM. Following transplantation, fasting and peak blood glucose levels were significantly higher in both patients with DM and PTDM patients compared with those without DM.

**Table 3:** Outcomes of transplant admission.

Outcomes of admission	No DM	DM	PTDM
Length of stay^#^	13.0 ± 5.5	16.9 ± 9.7^*∗*^	15.5 ± 6.4
AR (%)	7.6	13.8	25.9^*∗*^
Need for RRT (%)	19.6	48.3^*∗*^	51.9^*∗*^
Infective complications (%)	13.0	34.5^*∗*^	14.8

^#^Expressed as mean ± SD; ^*∗*^ *p* < 0.05 compared to no DM.

No DM: patients without DM, DM: patients with DM prior to transplantation, PTDM: posttransplant diabetes mellitus, AR: acute rejection, and RRT: renal replacement therapy.

Statistical analysis by ANOVA, chi-squared, and *t*-tests.

Following transplantation, DM patients had a longer hospital stay, had more infective complications, and were more likely to require renal replacement therapy. PTDM patients had increased rates of acute rejection and return to renal replacement therapy.

**Table 4:** Outcomes at 12 months following transplant.

Outcomes at 12 months	No DM	DM	PTDM
eGFR (mL/min/1.73 m^2^)^#^	45 ± 17	46 ± 15	45 ± 18
Need for RRT (%)	2.2	3.4	0.0
Number of rejection episodes^#^	0.3 ± 0.6	0.4 ± 0.6	0.3 ± 0.5
Cardiovascular complication (%)	4.3	20.7^*∗*^	3.7
Infection requiring admission (%)	31.5	41.4	37.0
Death (%)	2.2	10.3^∧^	0.0
RRT or death at one year (%)	4.3	10.3	0

^#^Expressed as mean ± SD; ^*∗*^ *p* < 0.05 compared to no DM; ^∧^ *p* = 0.06.

No DM: patients without DM, DM: patients with DM prior to transplantation, PTDM: posttransplant diabetes mellitus, and RRT: renal replacement therapy.

Statistical analysis by ANOVA, chi-squared, and *t*-tests.

At 12 months after transplant, only DM patients had increased rates of cardiovascular complications compared to those without DM.
